# Biogas production management systems with model predictive control of anaerobic digestion processes

**DOI:** 10.1007/s00449-020-02404-7

**Published:** 2020-07-18

**Authors:** Kazuto Yoshida, Naoto Shimizu

**Affiliations:** 1 Graduate School of Agriculture, Hokkaido University, Sapporo, Hokkaido 060-8589 Japan; 2 Agricultural Engineering Laboratory, Research Faculty of Agriculture, Hokkaido University, Kita 9 Nishi 9, Kita-ku, Sapporo, Hokkaido 060-8589 Japan

**Keywords:** Management system, Anaerobic digestion process, Prediction model, Feedstock determination, Numerical optimization

## Abstract

We developed a biogas production management system to control biogas production by determining the feedstock inputs to the anaerobic digestion process according to fluctuations of the renewable energy supply. The developed system consists of three functions: a prediction model for the anaerobic digestion processes, a parameter-estimation system, and a feedstock-determination controller. A prediction model for the anaerobic digestion processes in a state-space representation was constructed for the input–output relationship of biogas generation from organic compounds and the state of methane fermentation. A parameter-estimation system that estimated the parameters included in the prediction model from actual operating process data was built based on adaptive identification theory. The feedstock-determination controller was established based on model predictive control as a method to control biogas production. From the results of the identification experiment, the least square estimator of the parameters converged as the training data increased, and a reliable parameter was given in 1 week. From the results of the numerical simulation and the control experiment, it was confirmed that the biogas production management system developed in this study had a high prediction accuracy and control performance.

## Introduction

Increasing and stabilizing renewable energy supply, which will not be exhausted, is necessary and can be achieved with sustainable-development goals. However, current global energy consumption depends primarily on fossil fuels, which are becoming scarce. Thus, there is significant risk if all production activities ended due to resource depletion. Renewable energy output fluctuation due to varying environmental conditions, such as with wind and solar power, can inhibit the spread of renewable energy. For practical applications, backup via stable power sources, such as thermal or nuclear power, and energy carriers manufacturing, such as hydrogen, are required. In the future, it will be necessary to build an energy management system that leads to a stable supply of renewable energy without fossil fuels. Szarka et al. [1] and Hahn et al. [2] stated that biomass is one of the more robust renewable resources. It is impacted upon less by environmental change and has the potential for flexible power generation to compensate for such fluctuations. Biomass will play an important role in supply and demand adjustment in the renewable energy management system.

For energy production using biomass, anaerobic digestion of waste biomass is an excellent method that can simultaneously perform energy recovery and waste treatment [3, 4]. Numerical optimization of the anaerobic digestion process has been studied for various methods. Mendez-Acosta et al. regulated volatile fatty acid concentration and total alkalinity, both inhibitors of anaerobic digestion, to improve process stability using a dynamic model [5]. Mauky et al. [6] developed a feeding management strategy to compensate for the differences between energy supply and demand for the International Water Association (IWA) Anaerobic Digestion Model No. 1 (ADM1) [7]. During anaerobic digestion processes, if the feedstock input is changed rapidly, the fermentation state may shift excessively and inhibit fermentation [8, 9]. An improved model for predicting biogas generation while presuming and stabilizing the fermentation state is needed, and AMD1 is widely used because of its excellent fermentation state estimation. However, a simplified model with emphasis on practicality that can predict the fermentation state and biogas production is required. Hend et al. developed a simple model for biogas generation and conducted a parametric study for optimization of the model constants [10]. Furthermore, other research on modeling and process control for anaerobic digestion is discussed, ranging from classical feedback control to advanced model-based control methods [11–13]. In this research, we focus on the advanced model-based control method that can control output to a flexible set-point, because we expect the role in supply and demand adjustment in the renewable energy management system for biomass.

However, while there have been many attempts to control the anaerobic digestion process, in an average biogas plant, a certain amount of feedstock is introduced at regular intervals, and control of biogas production is not widespread because of the various operating conditions (i.e., type of feedstock biomass and the solid concentration and temperature of the digestate) and processing purposes (i.e., waste treatment or energy production) [9]. In particular, the difference in the substrate of feedstock and in the metabolic activity of the bacteria involved anaerobic digestion have a big influence on the reaction rate of biogas production [15]. Therefore, it is necessary to evaluate biological parameters related to substrate and bacteria in controlling the anaerobic digestion process. Some typical values for these biological parameters are given in ADM1 [7], but it is required to evaluate under more various conditions to realize the control of anaerobic digestion process. It is cumbersome and undesirable to determine all of the parameters experientially and arrange the control methods and concepts, because they are too complicated to easily manage. To control anaerobic digestion processes under various operating conditions and produce biogas to balance the difference between supply and demand, the following three functions are required: (1) a prediction model for biogas production and the unobserved fermentation state, (2) a parameter-estimation system that determines the parameters for each operating condition included in the model automatically, and (3) a feedstock-determination controller that determines the amount of feedstock so that biogas production stabilizes the renewable energy supply.

**Fig. 1:**
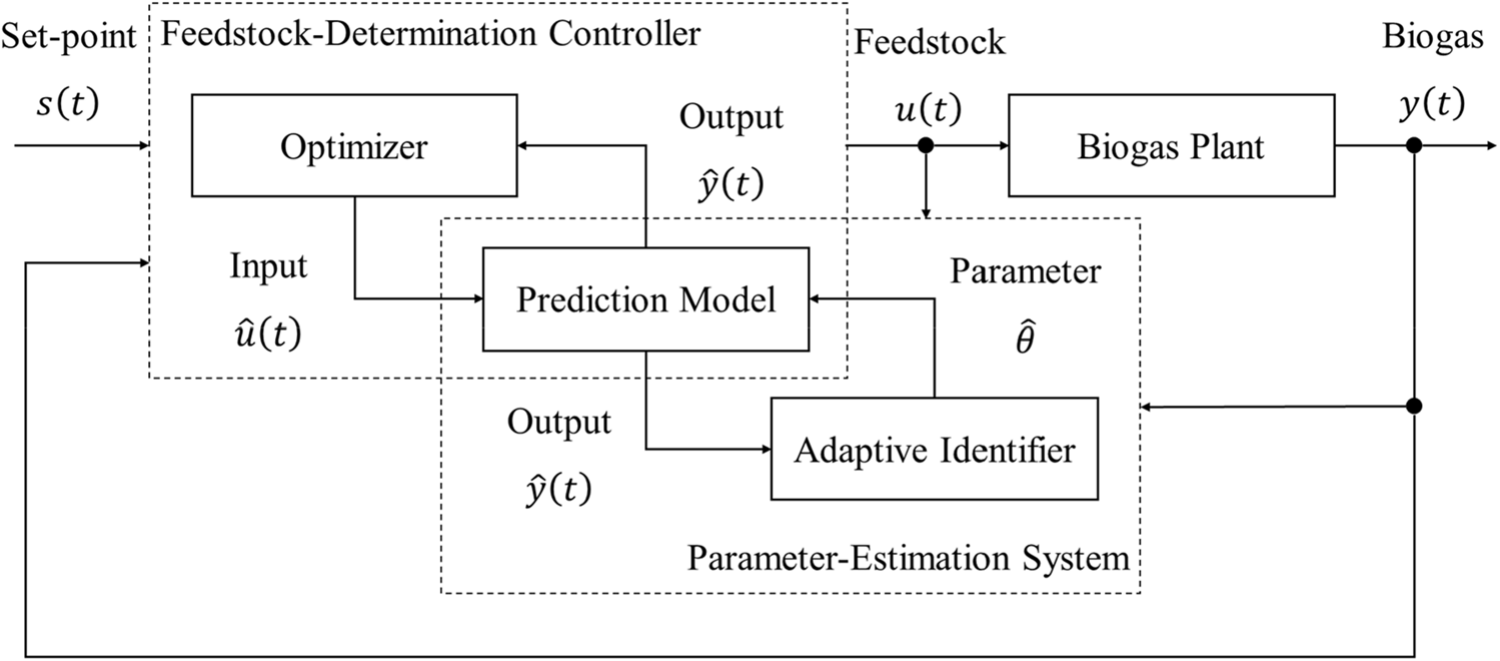
Biogas production management system with the prediction model of anaerobic digestion processes, the parameter-estimation system and the feedstock-determination controller

We proposed a biogas production management system with these three functions, as shown in Fig. 1. The aim of the present research was to develop these functions using the following methods: (1) developing a state-space representation that expressed the input–output relations to estimate unobservable states; (2) identifying the system identification theory that estimated parameters, automatically including the model from actual operating data of the process; and (3) modeling the predictive controls that optimized inputs so that outputs matched a future set-point while satisfying some constraints.

## Materials and methods

### Anaerobic digestion process flow

**Fig. 2:**
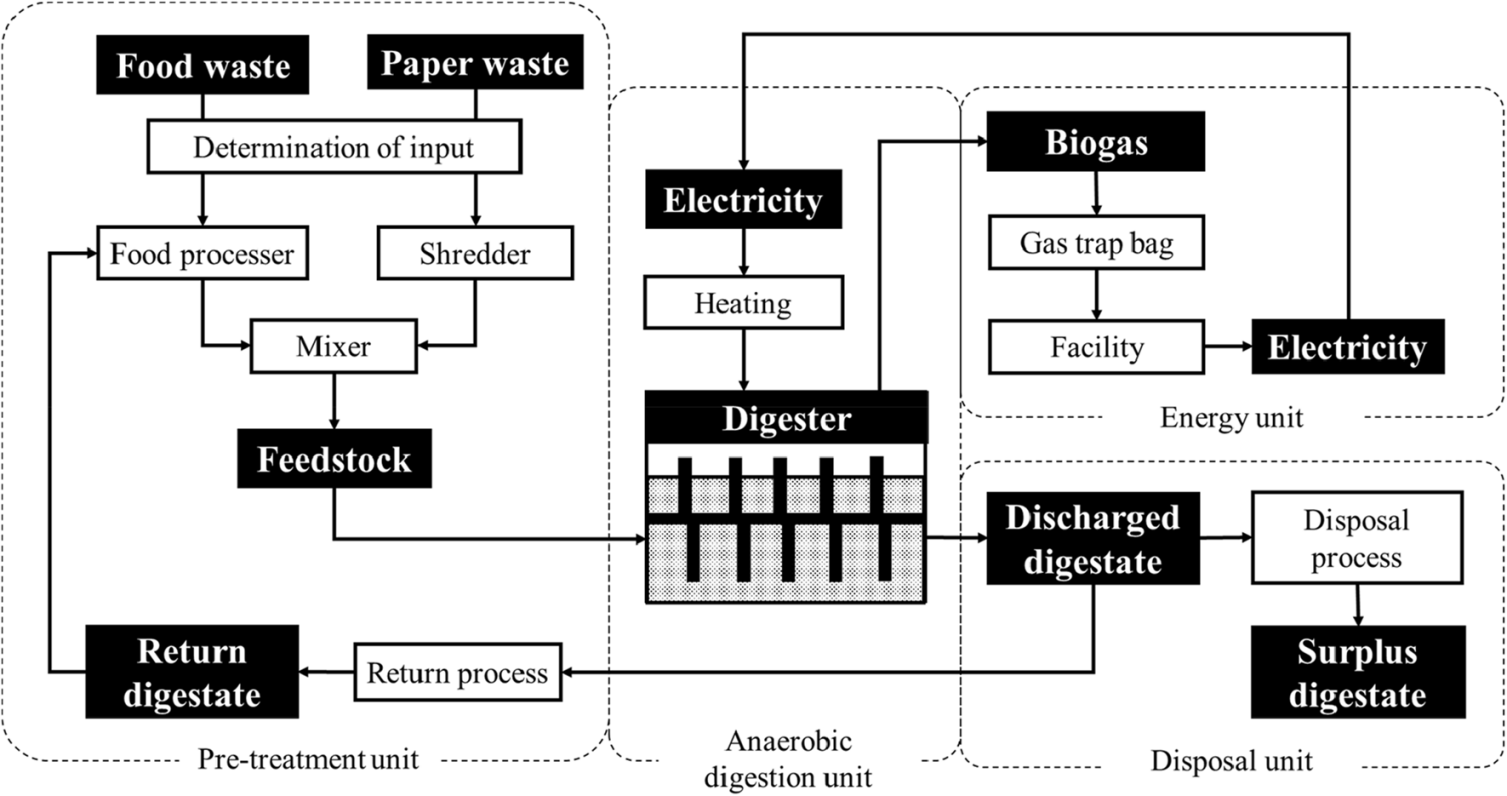
Anaerobic digestion processing flow. The feedstock prepared from food and paper waste in the pre-treatment unit was added to the anaerobic digester in the anaerobic digestion unit. Then, biogas was collected by a gas trap bag in the energy unit, and digestate was disposed in the disposal unit

The flow diagram for the anaerobic digestion process used in this study is shown in Fig. 2 [20]. Food waste ground with a food processor and discarded copy paper from Hokkaido University cut with a shredder were used as raw materials. Feedstock was made by mixing food waste (N-rich material) and paper waste (C-rich material) to adjust the C/N ratio to approximately 23, because N-rich feedstock can cause ammonia inhibition [14, 15]. Thus, the ground food and shredded paper waste were mixed in a mass ratio of 2.5:1. The mixed feedstock was added to a horizontal cylindrical reactor (effective volume of 0.235 m^3^) that was heated to approximately 52 °C and stirred regularly to degas and ensure proper mixing of the feedstock. Biogas was collected using a gas trap bag. A portion of the excreted digestate was collected at the time of feeding.

The total and volatile solids of the raw materials were approximately 40% and 35%, respectively. The digestate in the reactor was maintained at a thermophilic temperature (52 °C.). Therefore, the anaerobic digestion process used in this study was categorized as a dry thermophilic treatment, and no additional water was required [9].

### Prediction model of anaerobic digestion process

At first, we made several assumptions about the anaerobic digestion process to construct a simplified model.

1. The model dealt with the overall reaction from the decomposition of organic compounds (substrate) by bacteria to the production of biogas.
2. The reactor was semi-batch and had complete-mixing flow.
3. The volume of sludge in the reactor was constant at 200 L.

**Fig. 3:**
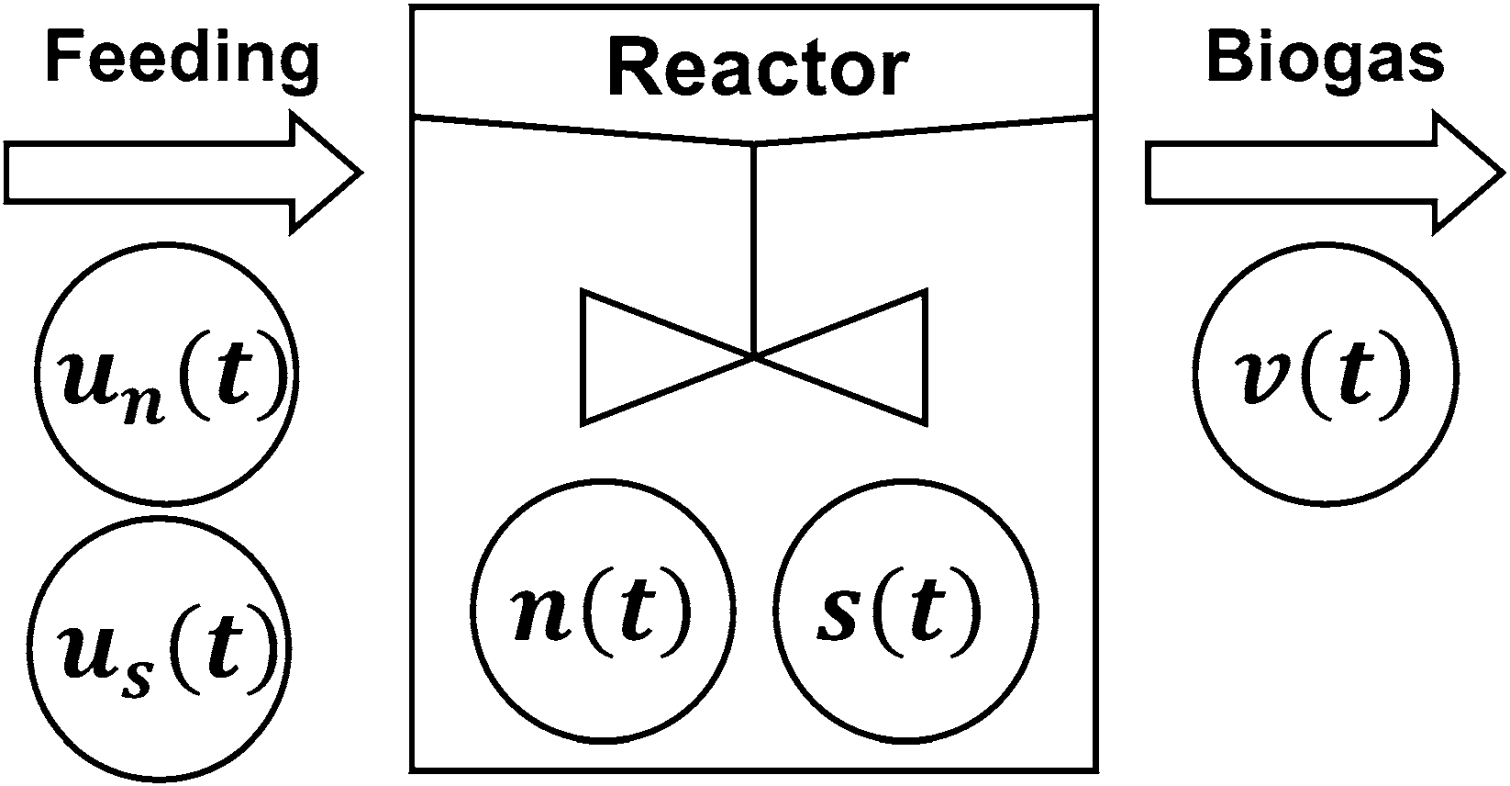
Representation of the semi-batch-type reactor used for anaerobic digestion in the present study. *u*_*n*_(*t*) bacteria input (g/L/h), *u*_*s*_(*t*) substrate input (g/L/h), *n*(*t*) bacteria concentration (g/L), *s*(*t*) substrate concentration (g/L), and *v*(*t*) biogas flow rate (L/h)

To describe the mathematical state of the process, we introduced five variables: (1) the bacterial concentration in the digestate “*n*(*t*)” and (2) the substrate concentration in the digestate “*s*(*t*)” as the state variables representing the fermentation state; (3) bacterial concentrations in the feedstock “*u*_*n*_(*t*)” and (4) substrate concentrations in the feedstock “*u*_*s*_(*t*)” were the manipulated variables; and (5) biogas production “*v*(*t*)” was the control variable. Using these factors, the reactions of the simplified anaerobic digestion process are shown in Fig. 3 [20].

The mathematical model based on the mass balance theory was given by concatenating two differential equations and one algebraic equation. The first represented bacterial growth, the second represented substrate decomposition, and the third represented biogas generation (Eq. 1). The bacterial growth equation was the logistic difference equation, an effective equation in the field of population biology [16]. The substrate decomposition and biogas generation equations showed that these occur with bacterial growth [10]. The specific growth rate in these equations was given by the modified Monod equation [17]:

1 $$ \left\{ {\begin{array}{*{20}c} {\frac{{{\text{d}}n\left( t \right)}}{{{\text{d}}t}} = \left( {\mu \left( s \right) - b} \right)n\left( t \right)\left( {1 - \frac{n\left( t \right)}{{n_{\hbox{max} } }}} \right) + u_{n} \left( t \right)} \\ {\frac{{{\text{d}}s\left( t \right)}}{{{\text{d}}t}} = - \frac{1}{Y}\mu \left( s \right)n\left( t \right) + u_{s} \left( t \right)} \\ {v\left( t \right) = \left( {k_{g1} b + k_{g2} \frac{1}{Y}\mu \left( s \right)} \right)mn\left( t \right)} \\ \end{array} } \right. $$

$$ \mu \left( s \right) = \mu_{\hbox{max} } \frac{s\left( t \right)}{{k_{s} + s\left( t \right) + k_{i} s^{2} \left( t \right)}}, $$

where $n\left( t \right)$ = bacterial concentration (kg/m^3^), $\mu \left( {\text{s}} \right)$ = specific growth rate (h^−1^), $b$ = autolysis rate (h^−1^), $n_{\hbox{max} }$ = maximum bacterial concentration (kg/m^3^), $u_{n} \left( t \right)$ = bacterial input (kg/m^3^/h), $s\left( t \right)$ = substrate concentration (kg/m^3^), $Y$ = bacterial cell yield, $u_{s} \left( t \right)$ = substrate input (kg/m^3^/h), $v\left( t \right)$ = biogas flow rate (m^3^/h), $m$ = sludge volume (m^3^), $k_{g1} , k_{g2}$ = biogas generation coefficients, $\mu_{\hbox{max} }$ = maximum specific growth rate (h^−1^), $k_{s}$ = dissociation constant (kg/m^3^), and $k_{i}$ = inhibition coefficient.

These biological parameters in Eq. (1) are important to describe the characteristics of the anaerobic digestion process. However, it is difficult to estimate all of them experientially under various operating conditions. To construct a feasible biogas production management system, they are aggregated into some parameters that represent the relationship between input and output. The linear time invariant state-space model was obtained by the perturbation method near the equilibrium point (Eq. 2). The matrices “*A*_p_, *B*_p_, *C*_p_”, called the Jacobian matrices, were partial derivative matrices evaluated by the equilibrium point. They were parameters that provided information relating to the characteristics of the anaerobic digestion process according to the operating conditions [i.e., substrate of feedstock “*s*(*t*)” and metabolic activity of bacteria “*n*(*t*)”]:

2 $$ \begin{aligned} \frac{{{\text{d}}\varvec{X}\left( t \right)}}{{{\text{d}}t}} & = \varvec{A}_{\text{p}} \varvec{X}\left( t \right) + \varvec{B}_{\text{p}} \varvec{U}\left( t \right), \\ y\left( t \right) & = \varvec{C}_{\text{p}} \varvec{X}\left( t \right) \\ \end{aligned} $$

where $$ \varvec{X}\left( t \right) = \left[ {\begin{array}{*{20}c} {n\left( t \right)} \\ {s\left( t \right)} \\ \end{array} } \right],  \varvec{U}\left( t \right) = \left[ {\begin{array}{*{20}c} {u_{n} \left( t \right)} \\ {u_{s} \left( t \right)} \\ \end{array} } \right],\varvec{  }y\left( t \right) = v\left( t \right), $$ $$ \varvec{A}_{\text{p}} = \left. {\frac{{\partial \varvec{F}}}{{\partial \varvec{X}\left( t \right)}}} \right|_{{\left( {\varvec{X}_{\text{eq}} ,\varvec{U}_{\text{eq}} ,t} \right)}} = \left[ {\begin{array}{*{20}c} {a_{11} } & {a_{12} } \\ {a_{21} } & {a_{22} } \\ \end{array} } \right],\quad \varvec{B}_{\text{p}} = \left. {\frac{{\partial \varvec{F}}}{{\partial \varvec{U}\left( t \right)}}} \right|_{{\left( {\varvec{X}_{\text{eq}} ,\varvec{U}_{\text{eq}} ,t} \right)}} = \left[ {\begin{array}{*{20}c} {b_{11} } & {b_{12} } \\ {b_{21} } & {b_{22} } \\ \end{array} } \right], $$

$$ \varvec{C}_{\text{p}} = \left. {\frac{\partial g}{{\partial \varvec{X}\left( t \right)}}} \right|_{{\left( {\varvec{X}_{\text{eq}} ,t} \right)}} = \left[ {\begin{array}{*{20}c} {c_{11} } & {c_{12} } \\ \end{array} } \right], $$

$$ \varvec{F}\left( {\varvec{X}\left( t \right),\varvec{U}\left( t \right),t} \right) = \left[ {\begin{array}{*{20}c} {f_{1} \left( {\varvec{X}\left( t \right),\varvec{U}\left( t \right),t} \right)} \\ {f_{2} \left( {\varvec{X}\left( t \right),\varvec{U}\left( t \right),t} \right)} \\ \end{array} } \right], $$

$$ f_{1} \left( {\varvec{X}\left( t \right),\varvec{U}\left( t \right),t} \right):\frac{{{\text{d}}n\left( t \right)}}{{{\text{d}}t}} = \left( {\mu \left( s \right) - b} \right)n\left( t \right)\left( {1 - \frac{n\left( t \right)}{{n_{\hbox{max} } }}} \right) + u_{n} \left( t \right), $$

$$ f_{2} \left( {\varvec{X}\left( t \right),\varvec{U}\left( t \right),t} \right): \frac{{{\text{d}}s\left( t \right)}}{{{\text{d}}t}} = - \frac{1}{Y}\mu \left( s \right)n\left( t \right) + u_{s} \left( t \right), $$

$$ g\left( {\varvec{X}\left( t \right),t} \right):v\left( t \right) = \left( {k_{g1} \frac{1}{Y}\mu \left( s \right) + k_{g2} b} \right)mn\left( t \right), $$

$$ a_{11} ,\;a_{12} ,\;a_{21} ,\;a_{22} ,\;b_{11} ,\;b_{12} ,\;b_{21} ,\;b_{22} ,\;c_{11} ,\;c_{12} = {\text{Jacobian elements}}. $$

The discrete input and output relational expressions were obtained by a *Z*-transformation on the state-space model, and the parameters in Eq. (2) were converted to “*a*_1,2_” and “*b*_1,2,3,4_” in Eq. (3). If their correct values were found, biogas generation “*y*(*k*)” could be predicted by bacterial and substrate concentrations in the feedstock “***U***(*k*)” in Eq. (3):

3 $$ a_{f} \left( q \right)y\left( k \right) = \varvec{B}_{f} \left( q \right)\varvec{U}\left( k \right), $$

where $$ a_{f} \left( q \right) = q^{ - 2} + \left( { - a_{11} - a_{22} } \right)q^{ - 1} + \left( {a_{11} a_{22} - a_{12} a_{21} } \right)q^{ - 2} + a_{1} q^{ - 1} + a_{2} , $$ and $$ \varvec{B}_{f} \left( q \right)\left[ {\begin{array}{*{20}c} {c_{11} \left( {q^{ - 1} - a_{22} } \right) + c_{12} a_{21} } & {c_{11} a_{12} + c_{12} \left( {q^{ - 1} - a_{11} } \right)} \\ \end{array} } \right] = \left[ {\begin{array}{*{20}c} {b_{1} q^{ - 1} + b_{2} } & {b_{3} q^{ - 1} + b_{4} } \\ \end{array} } \right]. $$

### Parameter-estimation system

**Fig. 4:**
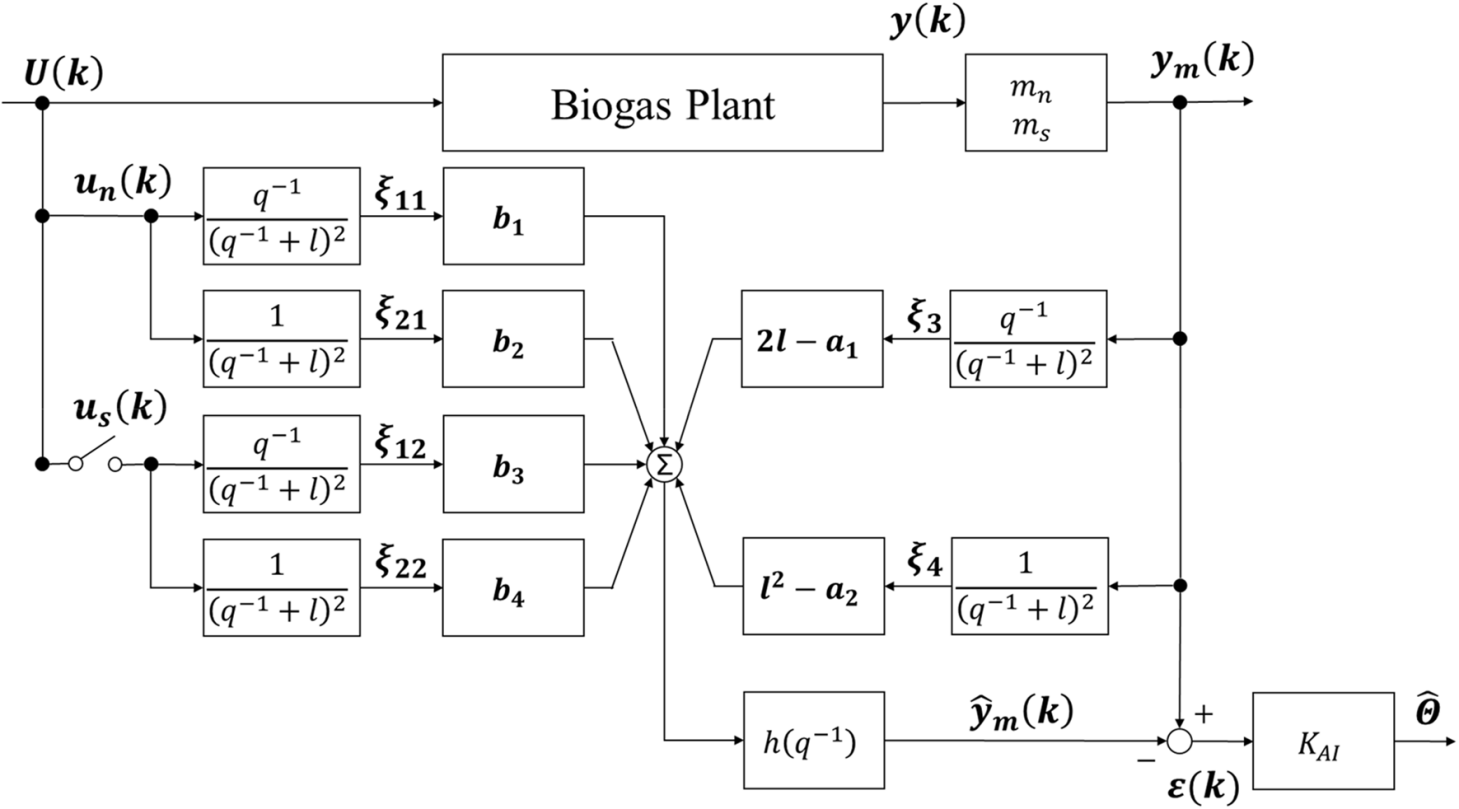
Adaptive identifier. ***U***(*k*) feedstock input (g/L/h), *u*_*n*_(*t*) bacteria input (g/L/h), *u*_*s*_(*t*) substrate input (g/L/h), *y*(*t*) biogas flow rate (L/h), *y*_*m*_(*t*) scaled biogas flow rate (L/h), *ξ*_11,12,21,22,3,4_, control signals, *a*_1,2_” and “*b*_1,2,3,4_ parameters, *h*(*q*^−1^) filter, *m*_*n*_ scaling coefficient related to bacterial output, *m*_*s*_ scaling coefficient related to substrate output, *l* control system design constant, $\hat{\varvec{y}}$_*m*_(*t*) predicted scaled biogas flow rate (L/h), *ε*(*k*) error (L/h), *K*_AI_ coefficient for least squares method, and $\hat{\varTheta }$ estimated parameters

We developed a parameter-estimation system using the adaptive identification theory to estimate these parameters from the actual operation data of the process [18]. The mechanism for the adaptive identifier, which was the core of the parameter-estimation system, is shown in Fig. 4 [20]. Input and output data were multiplied by the filter to provide the control signals “*ξ*_11,12,21,22,3,4_”, and the control signals were multiplied by the operation parameters and integrated to provide the output prediction. Then, the linear relationship between the output and the parameters with the proportionality constant “*ξ*_11,12,21,22,3,4_” was derived (Eq. 4). The parameters in Eq. (3) were integrated into “*Θ*” in Eq. (4):

4 $$ y\left( k \right) = h\left( {q^{ - 1} } \right)\varvec{\varTheta}^{T}\varvec{\varXi}\left( k \right), $$

where $$ \varvec{\varTheta}= \left[ {\begin{array}{*{20}c} {\begin{array}{*{20}c} {b_{1} } & {b_{3} } & {b_{2} } \\ \end{array} } & {\begin{array}{*{20}c} {b_{4} } & {2l - a_{1} } & {l^{2} - a_{2} } \\ \end{array} } \\ \end{array} } \right]^{T} , $$ $$ \varvec{\varXi}\left( k \right) = \left[ {\begin{array}{*{20}c} {\begin{array}{*{20}c} {\xi_{11} \left( k \right)} & {\xi_{12} \left( k \right)} & {\xi_{21} \left( k \right)} \\ \end{array} } & {\begin{array}{*{20}c} {\xi_{22} \left( k \right)} & {\xi_{3} \left( k \right)} & {\xi_{4} \left( k \right)} \\ \end{array} } \\ \end{array} } \right]^{T} , $$ and $$ l = {\text{control system design constant}} . $$.

The difference between the measured output value and the calculated value with the estimated parameters was defined as the output error “*ε*(*t*)” (Eq. 5):

5 $$ \varepsilon \left( k \right) = y\left( k \right) - h\left( {q^{ - 1} } \right)\hat{\varvec{\varTheta }}^{T}\varvec{\varXi}\left( k \right). $$

The least squares estimate of the parameter was obtained by the normal equation (Eq. 6), applying the least squares method to Eq. (5) with *n* data sets representing input and output. In this study, we updated the least squares estimator of the parameter each time a new data set was acquired. To avoid repetitively solving the inverse matrix operation of Eq. (6), which has a large computational load, the sequential least squares method was adopted:

6 $$ \hat{\varvec{\varTheta }} = \left( {\varvec{\varXi}_{n}\varvec{\varXi}_{n}^{T} } \right)^{ - 1}\varvec{\varXi}_{n} \varvec{Y}_{n}^{T} , $$

where $$ \varvec{\varXi}_{n} = \left[ {\begin{array}{*{20}c} {\begin{array}{*{20}c} {\varvec{\varXi}\left( 1 \right)} & {\varvec{\varXi}\left( 2 \right)} \\ \end{array} } & {\begin{array}{*{20}c} \cdots & {\varvec{\varXi}\left( n \right)} \\ \end{array} } \\ \end{array} } \right]^{T} , $$ and $$ \varvec{Y}_{n} = \left[ {\begin{array}{*{20}c} {\begin{array}{*{20}c} {y\left( 1 \right)} & {y\left( 2 \right)} \\ \end{array} } & {\begin{array}{*{20}c} \cdots & {y\left( n \right)} \\ \end{array} } \\ \end{array} } \right]^{T} . $$.

As shown in Fig. 4, there was a switch relating to substrate inputs in the adaptive identifier, because we considered the anaerobic digestion process to be a two-input/one-output system. This enabled the estimation of all parameters in Eq. (3), which represented input–output characteristics between bacterial input and biogas generation and between substrate input and biogas generation, either with or without substrate input. The filter “*h*(*q*^−1^)”, scaling coefficients “*m*_*n*_ and *m*_*s*_” and control system design constant “*l*” were tuned to obtain the desired estimation results.

Using the discrete input and output relational expressions (Eq. 3) given by the adaptive identifier, it was possible to predict the biogas generation from the feedstock input. Furthermore, since the purpose of this research was to control biogas production while presuming and stabilizing the fermentation state, we introduced the realization theory to derive a state-space model from the input and output model with minimum information loss.

First, the impulse response vector that was the *Z*-transform pair of the input and output model was defined as follows:

7 $$ \varvec{H}\left( k \right) = \left[ {\begin{array}{*{20}c} {h_{n} \left( k \right)} & {h_{s} \left( k \right)} \\ \end{array} } \right], $$

where $$ h_{n} \left( k \right) = {\text{impulse response of biogas generation from bacteria }}\left[ {{\text{Lh}}^{ - 1} } \right], $$ and $$ h_{s} \left( k \right) = {\text{impulse response of biogas generation from subsrate }}\left[ {{\text{Lh}}^{ - 1} } \right]. $$.

However, this impulse response vector could also be described by the coefficient matrix of the state-space model, as follows:

8 $$ \varvec{H}\left( k \right) = \left\{ {\begin{array}{*{20}c} {0, \left( {k = 0} \right)} \\ {\varvec{C}_{p} \varvec{A}_{p}^{k - 1} \varvec{B}_{p} , \left( {k > 0} \right)} \\ \end{array} } \right.. $$

A matrix, called Hankel matrix, was constructed using the impulse response vectors, whose values were almost 0, and the coefficient matrix of the state-space model, as follows:

9 $$ \varvec{H}_{n} = \left[ {\begin{array}{*{20}c} {\begin{array}{*{20}c} {\begin{array}{*{20}c} {\begin{array}{*{20}c} {\varvec{H}\left( 1 \right)} \\ {\varvec{H}\left( 2 \right)} \\ \end{array} } \\ {\begin{array}{*{20}c} \vdots \\ {\varvec{H}\left( n \right)} \\ \end{array} } \\ \end{array} } & {\begin{array}{*{20}c} {\begin{array}{*{20}c} {\varvec{H}\left( 2 \right)} \\ {\varvec{H}\left( 3 \right)} \\ \end{array} } \\ {\begin{array}{*{20}c} \vdots \\ 0 \\ \end{array} } \\ \end{array} } \\ \end{array} } & {\begin{array}{*{20}c} {\begin{array}{*{20}c} {\begin{array}{*{20}c} \cdots \\ \cdots \\ \end{array} } \\ {\begin{array}{*{20}c} \ddots \\ \cdots \\ \end{array} } \\ \end{array} } & {\begin{array}{*{20}c} {\begin{array}{*{20}c} {\varvec{H}\left( n \right)} \\ 0 \\ \end{array} } \\ {\begin{array}{*{20}c} \vdots \\ 0 \\ \end{array} } \\ \end{array} } \\ \end{array} } \\ \end{array} } \right]\left[ {\begin{array}{*{20}c} {\varvec{C}_{p} \varvec{B}_{p} } & {\varvec{C}_{p} \varvec{A}_{p} \varvec{B}_{p} } & \cdots & {\varvec{C}_{p} \varvec{A}_{p}^{n - 1} \varvec{B}_{p} } \\ {\varvec{C}_{p} \varvec{A}_{p} \varvec{B}_{p} } & {\varvec{C}_{p} \varvec{A}_{p}^{2} \varvec{B}_{p} } & \cdots & 0 \\ \vdots & \vdots & \ddots & \vdots \\ {\varvec{C}_{p} \varvec{A}_{p}^{n - 1} \varvec{B}_{p} } & 0 & \cdots & 0 \\ \end{array} } \right]. $$

Then, the Hankel matrix from the coefficient matrices of the state-space model was described as the product of an observable matrix and a reachable matrix by singular value decomposition, as follows:

10 $$ \varvec{H}_{n} = \left[ {\begin{array}{*{20}c} {\begin{array}{*{20}c} {\varvec{C}_{p} } \\ {\varvec{C}_{p} \varvec{A}_{p} } \\ \end{array} } \\ {\begin{array}{*{20}c} \vdots \\ {\varvec{C}_{p} \varvec{A}_{p}^{n - 1} } \\ \end{array} } \\ \end{array} } \right]\left[ {\begin{array}{*{20}c} {\begin{array}{*{20}c} {\varvec{B}_{p} } & {\varvec{A}_{p} \varvec{B}_{p} } \\ \end{array} } & {\begin{array}{*{20}c} \cdots & {\varvec{A}_{p}^{n - 1} \varvec{B}_{p} } \\ \end{array} } \\ \end{array} } \right] =\varvec{\varOmega}_{1:n}\varvec{\varGamma}_{1:n} = \varvec{U\varSigma }_{n} \varvec{V}^{T} , $$

where $$ \varvec{\varOmega}_{1:n} = {\text{observal matrix}},\varvec{\varGamma}_{1:n} = {\text{reachable matrix}},\; \varvec{U},\varvec{V} = {\text{orthogonal matrix}}, {\text{and }}\varvec{\varSigma}_{n} = {\text{diagonal matrix}} $$.

Singular value decomposition was given by solving an eigenvalue problem. In this study, this solution was the QR decomposition using Gram–Schmitt orthogonalization and the LU decomposition using the Claus method.

From the results of singular value decomposition, the coefficient matrices of the state-space model, which were the original parameters in Eq. (2), were obtained, as follows:

11 $$ \begin{aligned} \varvec{A}_{p} =\varvec{\varOmega}_{(1:n - 1)}^{\dag }\varvec{\varOmega}_{(2:n)} \hfill \\ \varvec{B}_{p} =\varvec{\varOmega}_{1} \hfill \\ \varvec{C}_{p} =\varvec{\varOmega}_{1,} \hfill \\ \end{aligned} $$

where $$ \varvec{\varOmega}_{1:n - 1}^{\dag } = \left( {\varvec{\varOmega}_{1:n - 1}^{T}\varvec{\varOmega}_{1:n - 1} } \right)^{ - 1}\varvec{\varOmega}_{1:n - 1}^{T} . $$.

To quantitatively evaluate the prediction accuracy of the model with the estimated parameters, the goodness-of-fit index (GFI) was introduced (Eq. 7) [19]:

12 $$ {\text{GFI }}\left[ {\text{\% }} \right] = 100\left( {1 - \frac{{y\left( k \right) - \hat{y}\left( k \right)}}{{y\left( k \right) - {\text{mean}}\left( y \right)}}} \right). $$

### Feedstock-determination controller

We developed a feedstock-determination controller using model predictive control so that biogas production could stabilize the renewable energy supply. Model predictive control is a control theory that predicts future output and optimizes input so that it matches the set-point. When a set-point for the output is given to a plant, the set-point trajectory and a reference trajectory that is an ideal transition to the set-point are determined. The purpose of this study was to control the production of biogas to stabilize renewable energy supply, but we also gave a pseudo set-point in this paper. Biogas generation was 22 L/h from 0 to 120 h and then 0 L/h when the set-point trajectory and a reference trajectory that linearly changed 48 h after the set-point change were as follows:

13 $$ \begin{aligned} s\left( k \right) = \left\{ {\begin{array}{*{20}c} {y\left( k \right) = 22 \left( {k \le 120} \right)} \\ {y\left( k \right) = 0 \left( {k > 120} \right)} \\ \end{array} } \right. \hfill \\ r\left( k \right) = \left\{ {\begin{array}{*{20}c} {y\left( k \right) = 0.46k \left( {k < 48} \right)} \\ {y\left( k \right) = 22 \left( {48 \le k \le 120} \right)} \\ {y\left( k \right) = - 0.46k \left( {k > 120} \right)} \\ \end{array} } \right., \hfill \\ \end{aligned} $$

where $$ s\left( k \right) = {\text{set}} - {\text{point trajectory}}, {\text{and }}r\left( k \right) = {\text{reference trajectory}}. $$.

The following vectors for the variables in the control and predictive horizons were defined as follows:

14 $$ \begin{aligned} \varvec{Z}_{\text{MPC}} \left( k \right) = \left[ {\begin{array}{*{20}c} {\hat{z}\left( {k + H_{w} } \right)} \\ \vdots \\ {\hat{z}\left( {k + H_{p} } \right)} \\ \end{array} } \right] \hfill \\\varvec{\varGamma}\left( k \right) = \left[ {\begin{array}{*{20}c} {r\left( {k + H_{w} } \right)} \\ \vdots \\ {r\left( {k + H_{p} } \right)} \\ \end{array} } \right] \hfill \\ \Delta \varvec{U}_{\text{MPC}} \left( k \right) = \left[ {\begin{array}{*{20}c} {\Delta \hat{\varvec{U}}\left( {k + H_{w} } \right)} \\ \vdots \\ {\Delta \hat{\varvec{U}}\left( {k + H_{u} - 1} \right)} \\ \end{array} } \right], \hfill \\ \end{aligned} $$

where $$ \hat{z}\left( k \right) = {\text{output prediction}}, H_{w} = {\text{window parameter}},H_{u} = {\text{control horizon}} $$, $$ H_{p} = {\text{predictive horizon}},\;\varvec{Z}_{\text{MPC}} \left( k \right) = {\text{output prediction vector}} $$, $$ \varvec{\varGamma}\left( k \right) = {\text{reference trajectory vector}},{\text{and  }}\Delta \varvec{U}_{\text{MPC}} \left( k \right) = {\text{input change vector}} . $$.

The relationship among the vectors in Eq. (14) focused on output predictions and could be described using a state-space model as follows:

15 $$ \varvec{Z}_{\text{MPC}} \left( k \right) = \varvec{\varPsi X}\left( k \right) + \varvec{\varUpsilon U}\left( {k - 1} \right) + \varvec{T}\Delta \varvec{U}_{\text{MPC}} \left( k \right), $$

where $$ \varvec{\varPsi},\varvec{\varUpsilon},\varvec{T} = {\text{prediction matrix by state space model}} . $$.

The tracking error, which was the difference between the future reference trajectory and the free response, was defined by the following equation:

16 $$ \varvec{E}_{\text{MPC}} \left( k \right) =\varvec{\varGamma}\left( k \right) - \varvec{\varPsi X}\left( k \right) - \varvec{\varUpsilon U}\left( {k - 1} \right), $$

where $$ \varvec{E}_{\text{MPC}} \left( k \right) = {\text{tracking error vector}} . $$.

The control objective for the model predictive control in this study was to determine the minimum input when output matched the reference trajectory. Therefore, the evaluation function “*V*(*k*)” was given by adding the deviation between the future output of the plant and the reference trajectory and the sum of the inputs as the evaluation targets. The weight matrices “***Q***” and “***R***” were adjusted by the user according to the control purpose:

17 $$ V\left( k \right) = \varvec{Z}_{\text{MPC}} \left( k \right) -\varvec{\varGamma}\left( k \right)_{\varvec{Q}}^{2} + \Delta \varvec{U}_{\text{MPC}} \left( k \right)_{\varvec{R}}^{2} . $$

The optimal future input change was obtained using the least square method so that the evaluation function was minimized. Then, the feasible feedstock input amount was determined by integrating the optimal input at a certain time.

**Fig. 5:**
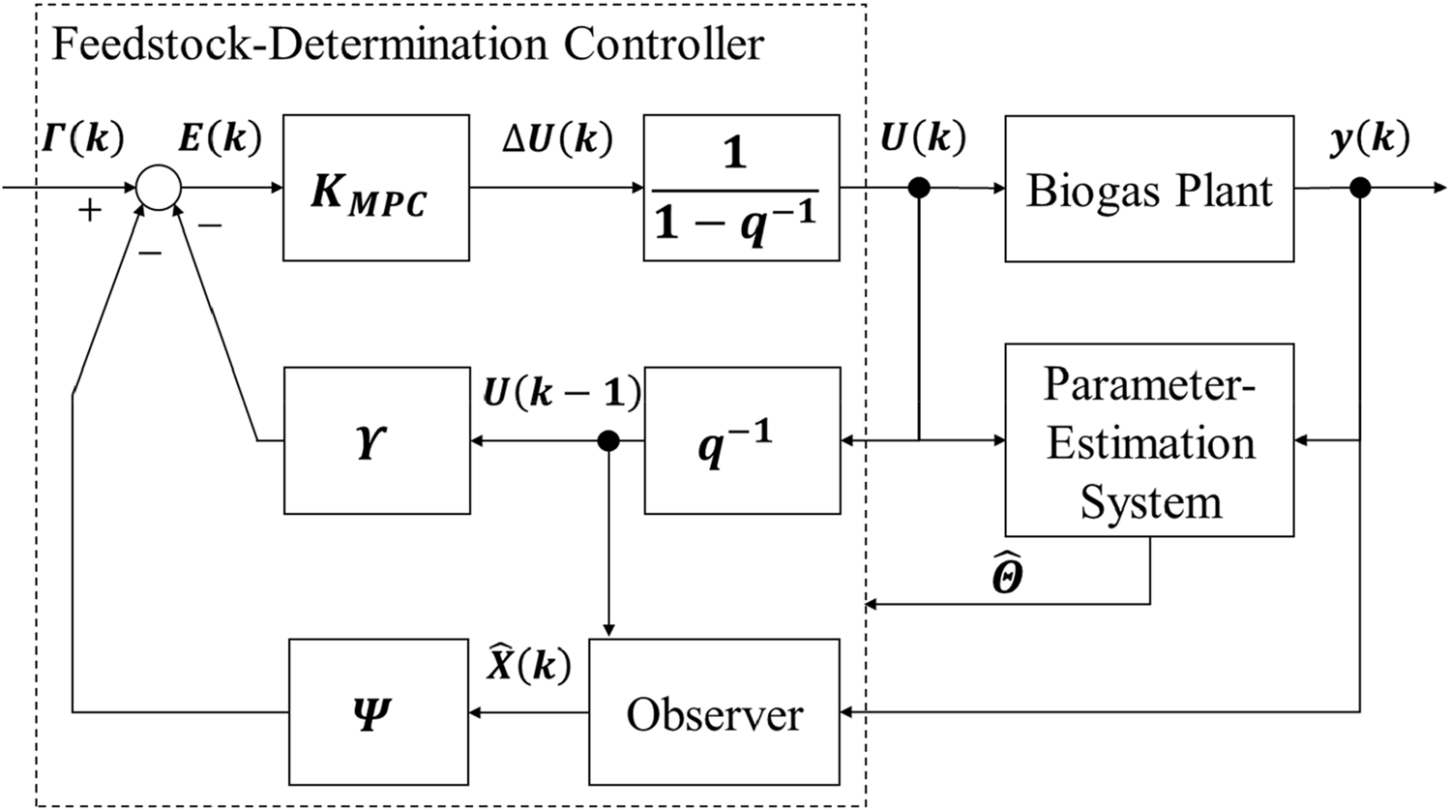
Feedstock-determination controller based on model predictive control. ***U***(*k*) feedstock input (g/L/h), *y*(*k*) biogas flow rate (L/h), $\hat{X}$(*k*) predicted state (g/L), *γ* prediction matrix by state-space model, ***ψ*** prediction matrix by state-space model, ***Γ***(*k*) reference trajectory vector, ***E***(*k*) tracking error vector, ∆***U***(*k*) input change vector and ***K***_MPC_ coefficient for least squares method

Figure 5 shows the structure of the feedstock-determination controller constructed from the results of the above formulation. The tracking error was given by taking the difference between the sum of the estimated state variables “***X***(*k*)” and the input data “***U***(*k*)” multiplied by the prediction matrix and the reference trajectory “***Γ***(*k*)”. The evaluation function was the sum of the output error and the input, and the optimal future input change was determined by the least square method “***K***_MPC_” so that it became the minimum. Furthermore, it was integrated at a certain time to obtain an executable input.

To evaluate control performance, we introduced mean square error (RMSE) focusing on output deviation, as follows:

18 $$ {\text{RMSE }} = \sqrt {\frac{1}{n}\mathop \sum \limits_{i = k}^{n} \left( {y\left( k \right) - r\left( k \right)} \right)^{2} } . $$

### Simulation data

Substrate concentration was defined as the mass reduction that occurred following heating at 105 °C for 24 h then 600 °C for 3 h in an oven. Biogas generation was measured hourly with a wet gas meter (W-NKDa-0.5B, SHINAGAWA) and recorded using a data logger (Data mini LR 5000, HIOKI).

**Fig. 6:**
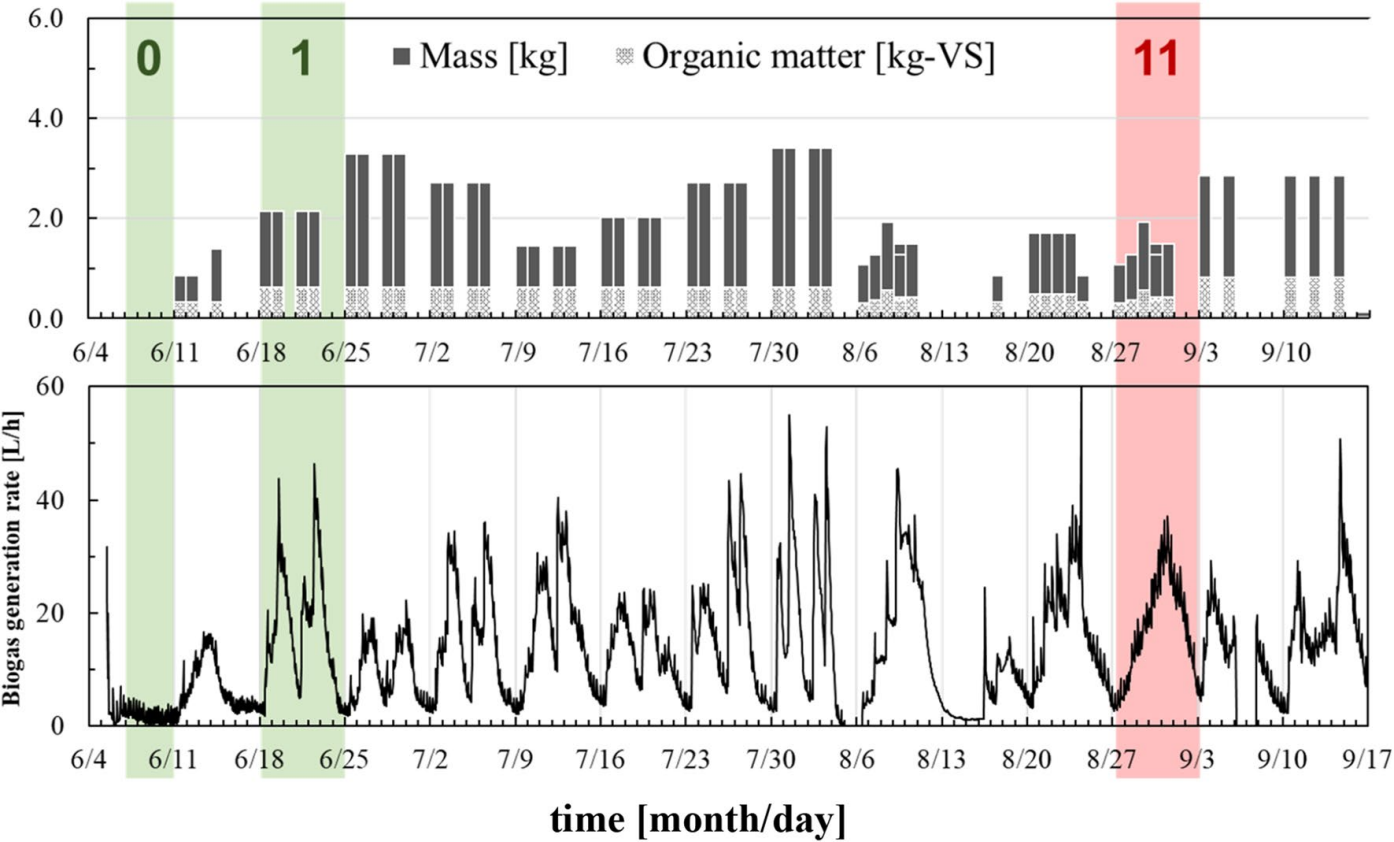
Simulation data: (I) feedstock input and (II) biogas generation rate

The data used in the simulation was the actual operation data collected by our laboratory in 2018, as shown in Fig. 6. Bacterial input at 0 h was taken as the amount of substrate contained in the digestate at the beginning of the process, and feedstock input was performed according to each operating condition. Since this study aimed an independent biogas plant feeding municipal solid waste, the bacterial input is only the first time. The data set for model construction to estimate the parameters was term 0, which was 72 h from 6/7 to 6/10 when no feedstock was loaded, and term 1, which was 168 h from 6/18 to 6/25 when full-scale feedstock was loaded. The control period was term 11 and was 168 h from 8/27 to 9/3 when the feedstock was determined by the biogas production management system, and the conventional input period was term 13, which was 168 h from 9/10 to 9/17 when the feedstock was loaded in a constant amount and at regular intervals. The organic loading rates of the model construction, the control and conventional periods were the same, 1.5 g-VS/L-digester/day. Sampling time for each date was 1 h that is a realizable value in the actual biogas plant.

## Results and discussion

**Fig. 7:**
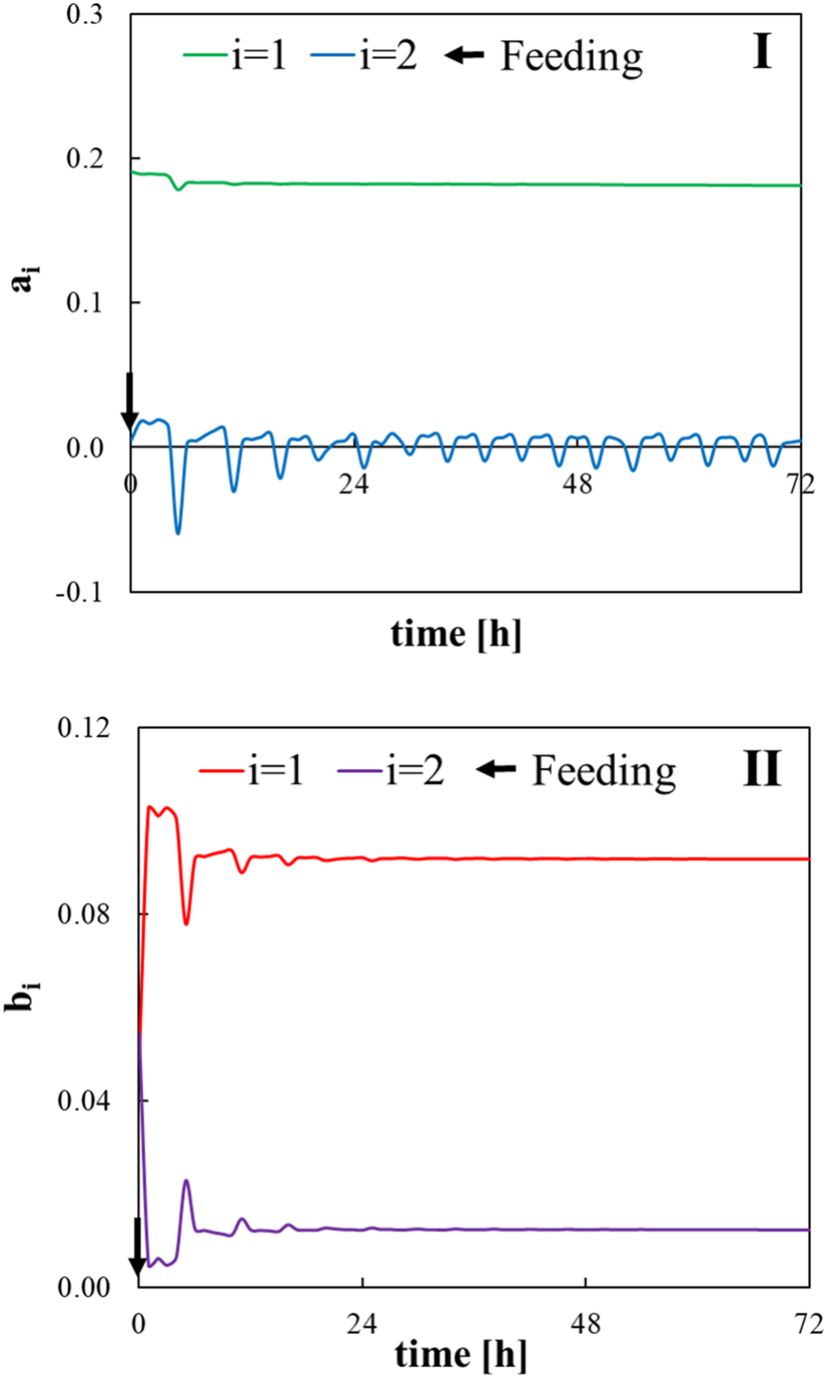
Estimated parameters related to bacterial input. “*a*_*i*_” means parameter on the output side in (Eq. 3) and “*b*_*i*_” means parameter on the input side in (Eq. 3). The numbers in the figure legend refer to the subscripts of the parameters “i”

**Fig. 8:**
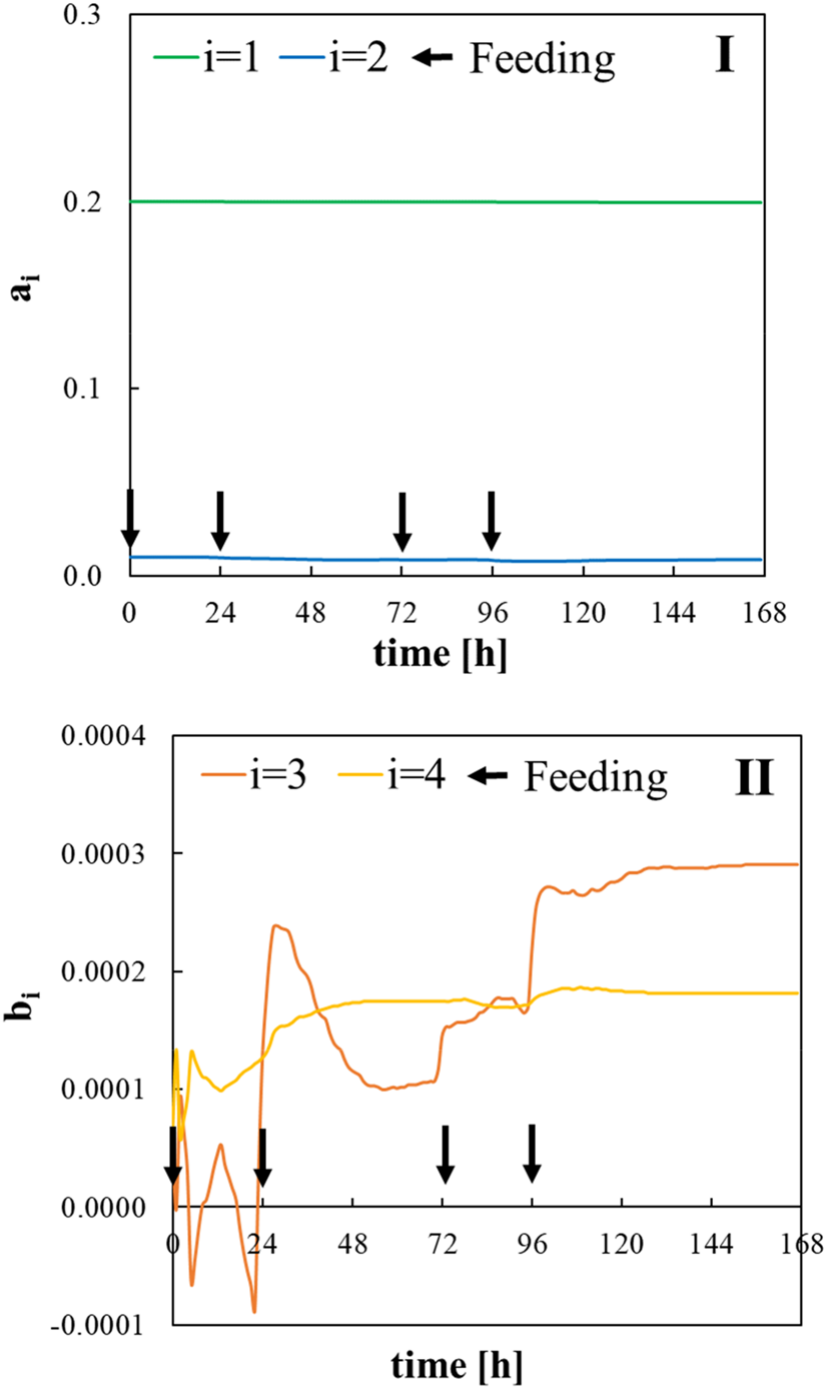
Estimated parameters related to substrate input. “*a*_*i*_” means parameter on the output side in (Eq. 3) and “*b*_*i*_” means parameter on the input side in (Eq. 3). The numbers in figure legend refer the subscripts of the parameters “*i*”

The results of tuning the constants related to the control system are shown in Table 1. Of these tuned constants, scaling coefficients have an impact on identification performance (filter and system design constant only have some relation to the convergence speed). Tuning of scaling coefficient related to substrate output is important, and biogas production from the substrate was the contribution of 10^4^ in this process. The results of parameter estimation using data from term 0 and 1 are shown in Figs. 7 and 8, respectively. The least square estimator of parameters converged as feedings were performed, and the training data increased and converged with approximately 24 h data for the bacterial input and approximately 144 h for the substrate input. Therefore, it was found that a reliable estimator was given by an identification experiment for more than 1 week. When operating conditions such as type of feedstock biomass are changed and there is a disturbance in the process, it is important to perform process control while confirming the degree of convergence of these parameters. A detailed review of the parameter-estimation system was previously reported [20].

**Table 1:** Tuned constants: *h*(*q*^−1^) filter, *m*_*n*_ scaling coefficient related to bacterial output, *m*_*s*_ scaling coefficient related to substrate output, and *l* system design constant

*m*_*n*_	*m*_*s*_	*l*	*h*(*q*^−1^)
1	0.0001	0.1	1

The state-space model (Eq. 2) obtained by realization theory was as follows:

19 $$ \begin{aligned} \frac{{{\text{d}}\varvec{X}\left( t \right)}}{{{\text{d}}t}} & = \varvec{A}_{p} \varvec{X}\left( t \right) + \varvec{B}_{p} \varvec{U}\left( t \right) \\ y\left( t \right) & = \varvec{C}_{p} \varvec{X}\left( t \right), \\ \end{aligned} $$

where $$ \varvec{A}_{p} = \left[ {\begin{array}{*{20}c} {0.9691} & {0.02027} \\ { - 0.1481} & {0.8443} \\ \end{array} } \right],    \varvec{B}_{p} = \left[ {\begin{array}{*{20}c} {0.003719} & {0.2457} \\ {0.009624} & {0.5145} \\ \end{array} } \right] $$, and $$ \varvec{C}_{p} = \left[ {\begin{array}{*{20}c} {29.98} & { - 8.590} \\ \end{array} } \right]. $$.

**Fig. 9:**
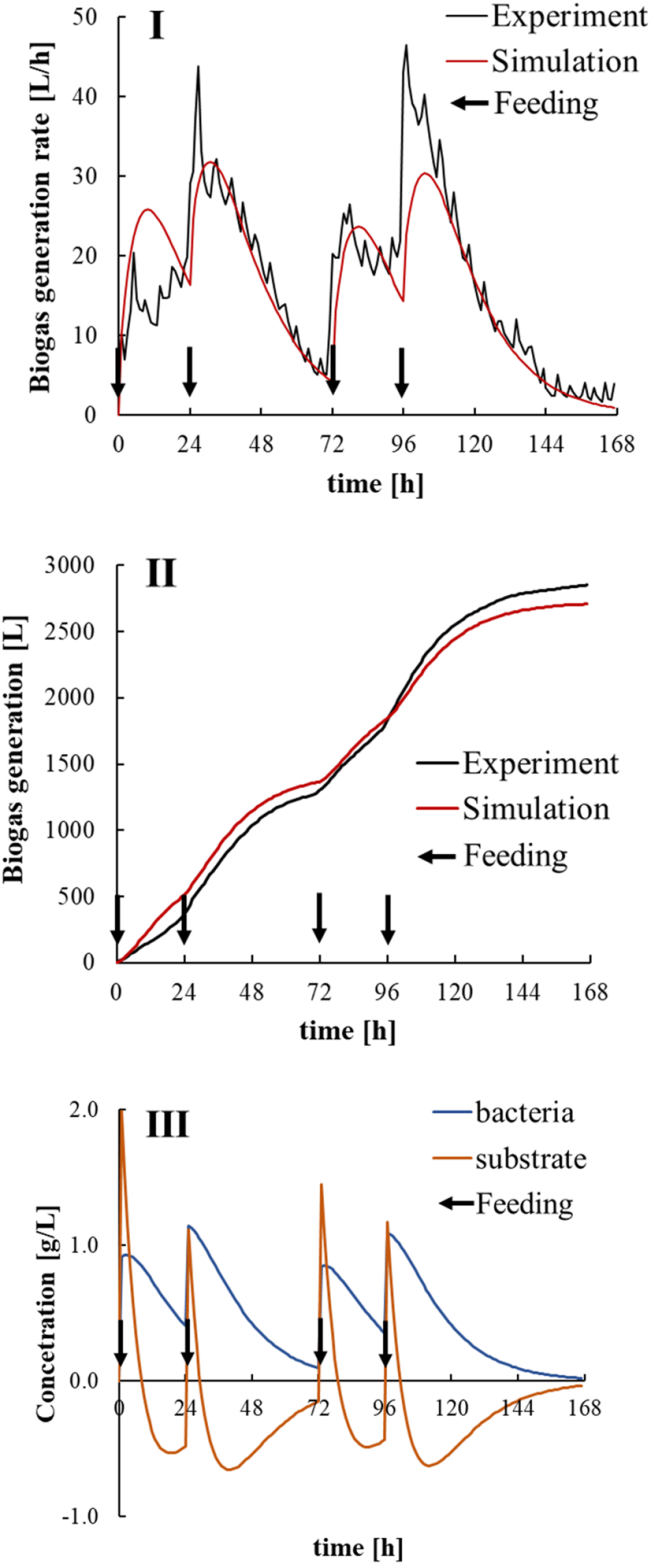
Numerical simulation result of anaerobic digestion processes for the data set that was used for model construction: (I) biogas generation rate, (II) accumulated biogas generation and (III) predicted fermentation state

Figure 9 shows the results of a numerical simulation for the model-construction data set using the constructed state-space model and estimated parameters. The GFI of the biogas generation rate was 46.1%, and the GFI of the accumulated biogas generation was 89.0%, indicating that the prediction model and the parameter-estimation system developed in this study have high prediction accuracy. The predicted biogas generation exhibited lower GFI than accumulated biogas generation because of the influence of degassing due to agitation and heating at regular frequency. A future issue to consider is the effects of biogas generation by degassing in the model. After the feedstock was added, the bacteria concentration slightly increased and then decreased slowly, and the substrate concentration decreased rapidly and then increased gradually. The fermentation state variables both fell to 0 after approximately 96 h. This result suggested that the bacterial concentration in this model represented the activity of the methanogens, and the substrate concentration represented the substrate required for methane production. It was conjectured that the methane fermentation did not proceed when the bacteria concentration was low, and the methanogen was in a deficient state when the substrate concentration was negative. Feeding strategies avoiding these states would be required to keep the process working as needed.

The model and the parameter-estimation system could automatically predict the biogas generation and fermentation states of the anaerobic digestion processes under various operating conditions. Furthermore, they were suitable for model predictive control, because its control performance greatly depended on the prediction accuracy of the model. In addition, the advantage of the parameter-estimation system was that the numerical simulation could be performed while updating parameters as the biogas plant was operating. In an actual biogas plant, it is necessary to consider that some operating conditions, such as feedstock composition and metabolic activity of the bacteria, change continually. Estimated parameters were adaptively identified in response to changes in operating conditions by the parameter-estimation system with a variable that limited using data. For example, in case of this process, the estimated parameters will reflect current feedstock composition and metabolic activity of the bacteria by limiting data to the lastest 144 h in this process.

**Fig. 10:**
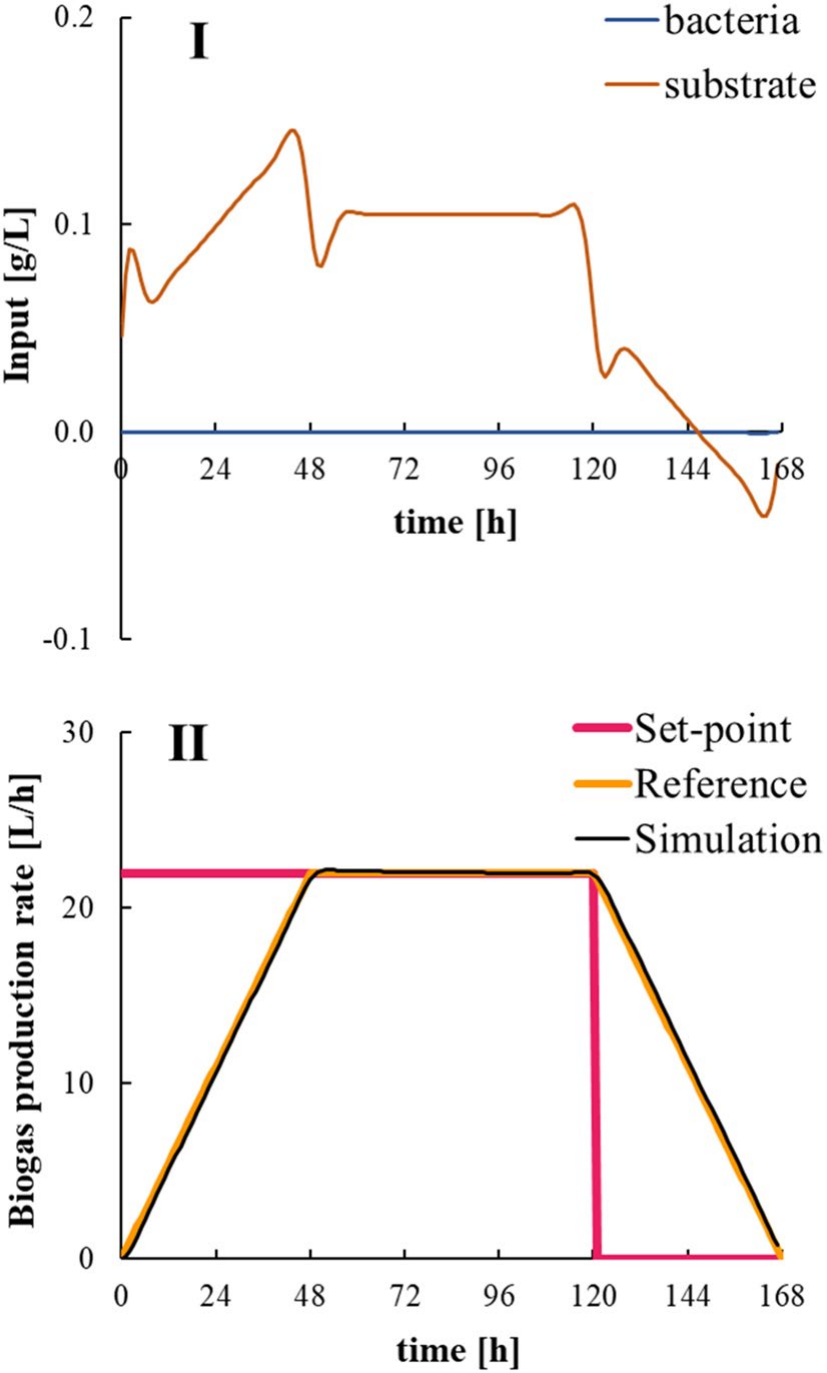
Numerical simulation result of the optimal solution given by model predictive control: (I) optimal feedstock input and (II) predicted biogas production rate

The results of the numerical simulation for the optimal solution given by model predictive control, i.e., biogas production rate, are shown in Fig. 10, where the RMSE was 0.317. To obtain the same control performance at all times and to avoid bacterial input, the weight matrices “***Q***” and “***R***” were tuned as follows:

20 $$ \varvec{Q} = \left[ {\begin{array}{*{20}c} 1 & 0 \\ 0 & 1 \\ \end{array} } \right],\quad \varvec{R} = \left[ {\begin{array}{*{20}c} {100} & 0 \\ 0 & {10} \\ \end{array} } \right]. $$

**Fig. 11:**
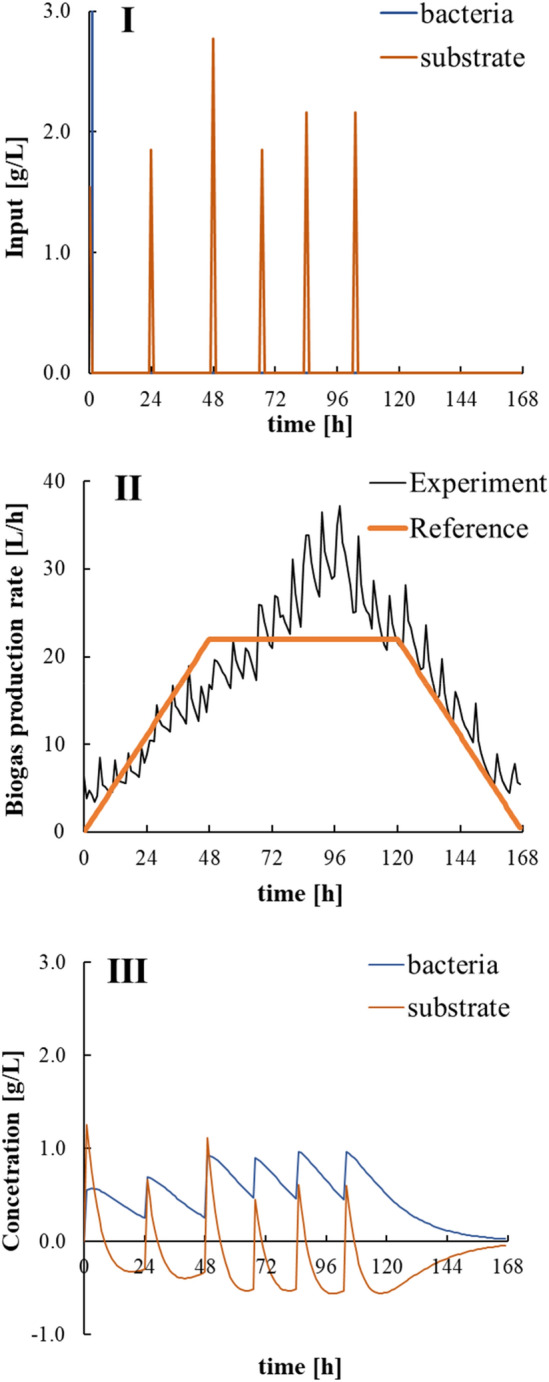
Numerical simulation result of the feasible solution given by model predictive control: (I) feasible feedstock input and (II) predicted biogas production rate

Figure 10 demonstrates that the feedstock-determination controller developed in this study gave the optimal solution to control biogas production with very good performance. Then, this optimal solution for feedstock input was integrated at discrete times with the constraint that the deviation of the biogas production rate was 5 or less at 48 to 120 h, and a feasible solution was determined, where the RMSE was 4.97. The results of the control period when the optimized feedstock was loaded is shown in Fig. 11. Regarding the prediction accuracy of the model, the GFI of the biogas production rate was 27.3% during the control period, and the GFI of the accumulated biogas production was 81.9% during the control period. There was no significant decrease compared with the GFI of the model-construction data; therefore, the model demonstrated high prediction accuracy during the control period. From the numerical simulation results, during the control period, the bacterial concentration was high, and the substrate concentration was low. As a result, the biogas production rate could be stabilized. As can be seen from the other periods in Fig. 6, even when a constant amount of feedstock was added at regular intervals, the biogas production rate did not remain stable. However, during the control period when the biogas production management system developed in this study was implemented, the biogas production rate remained stable following the reference trajectory. The calculated RMSE for the control period was 4.63. Although it was lower than the optimal solution of model predictive control, the biogas production management system could control the biogas production rate well throughout the control period.

## Conclusions

In this study, we developed a biogas production management system consisting of three functions: (1) the prediction model of anaerobic digestion, (2) the parameter-estimation system, and (3) the feedstock-determination controller, as shown in Fig. 1. The prediction model for anaerobic digestion processes was based on the mass balance theory, the parameter-estimation system was based on the adaptive identification theory, and the feedstock-determination controller was based on model predictive control. It was confirmed by the results of the numerical simulation and the control experiment that the biogas production management system had high prediction accuracy and control performance. A future issue to address is the stabilization of the supply of renewable energy by controlling biogas production to compensate for the difference between supply and demand using this system.
